# Use of virtual monoenergetic images for reduction of extensive dental implant associated artifacts in photon-counting detector CT

**DOI:** 10.1038/s41598-023-50926-3

**Published:** 2024-01-04

**Authors:** Yannik C. Layer, Narine Mesropyan, Patrick A. Kupczyk, Julian A. Luetkens, Alexander Isaak, Tatjana Dell, Benjamin P. Ernst, Ulrike I. Attenberger, Daniel Kuetting

**Affiliations:** 1 Department of Diagnostic and Interventional Radiology, University Hospital Bonn, Venusberg-Campus 1, 53127 Bonn, Germany; 2 Department of Otorhinolaryngology, University Hospital Bonn, Venusberg-Campus 1, 53127 Bonn, Germany

**Keywords:** Medical imaging, Computed tomography

## Abstract

Aim of this study was to assess the impact of virtual monoenergetic images (VMI) on dental implant artifacts in photon-counting detector computed tomography (PCD-CT) compared to standard reconstructed polychromatic images (PI). 30 scans with extensive (≥ 5 dental implants) dental implant-associated artifacts were retrospectively analyzed. Scans were acquired during clinical routine on a PCD-CT. VMI were reconstructed for 100–190 keV (10 keV steps) and compared to PI. Artifact extent and assessment of adjacent soft tissue were rated using a 5-point Likert grading scale for qualitative assessment. Quantitative assessment was performed using ROIs in most pronounced hypodense and hyperdense artifacts, artifact-impaired soft tissue, artifact-free fat and muscle tissue. A corrected attenuation was calculated as difference between artifact-impaired tissue and tissue without artifacts. Qualitative assessment of soft palate and cheeks improved for all VMI compared to PI (Median PI: 1 (Range: 1–3) and 1 (1–3); e.g. VMI_130 keV_ 2 (1–5); *p* < 0.0001 and 2 (1–4); *p* < 0.0001). In quantitative assessment, VMI_130 keV_ showed best results with a corrected attenuation closest to 0 (PI: 30.48 ± 98.16; VMI_130 keV_: − 0.55 ± 73.38; *p* = 0.0026). Overall, photon-counting deducted VMI reduce the extent of dental implant-associated artifacts. VMI of 130 keV showed best results and are recommended to support head and neck CT scans.

## Introduction

Dental implant associated artifacts in CT scans pose a common challenge in the assessment of the craniomandibular region. As lifestyle and dental medicine changed over the last decades, dental implants and fillings are frequently used and therefore are found in most CT examinations, especially in older patients. In a study among emergency patients from 2019 dental artifacts were present in 75% of CT scans^1^. These artifacts can severely hamper diagnostic assessment of CT scans and disguise tumor metastasis or overlap tumor delineation. Inflammation foci and traumatic injuries of the dental apparatus might be missed. As CT is the standard imaging modality for assessment of head and neck tumors as well as facial/cranial injuries and inflammatory diseases there is a strong need for dental implant associated artifact reduction to allow for diagnostic assessment, especially of the oral cavity^2^.

Most dental implant associated artifacts result from beam hardening, photon starvation and scattering^3^ and lead to reduced diagnostic quality of the adjacent soft tissue. The extent of these are influenced by various parameters such as material of the implants, tube voltage and current as well as reconstruction parameters^4^. Several studies employing dual energy CT systems based on energy integrating detectors have shown that the reconstruction of virtual monoenergetic images (VMI) can help to decrease the extent of implant associated artifacts^2,5–8^.

The new emerging technology of photon-counting detector CT (PCD-CT) has raised high expectations^9^. PCD-CT utilizes a direct conversion of X-ray photons into electronic signal, whereas energy-integrating detector CT (EID-CT) converts X-ray photons in a preceding step into visible light before the visible light is converted into an electronic signal through photo diodes. In recent studies, the inert advantages of photon-counting detector systems (i.e. lower image noise, higher SNR, higher CNR as well as higher spatial resolution with the potential for simultaneous dose reduction) have been demonstrated^10–17^. Photon-counting CT technology enables lower image noise, spectral information and exact photon energy arrangement, especially of low energy-photons, ultimately leading to the reconstruction of less artifact-burdened images. A phantom study showed a significant reduction in metal artifacts for orthopedic prosthesis compared to EID-CT^18^. Therefore, PCD-CT is expected to reduce artifacts compared to EID-CT^17^.

The aim of this study was to assess the clinical use of VMI for dental artifact reduction in PCD-CT as compared to a conventionally, reconstructed polychromatic image (PI).

## Materials and methods

The local institutional review board of the University Hospital Bonn (Ethics Committee of the Medical Faculty of the University of Bonn, Application number 159/22) approved this study. Informed consent was waived by the Ethics Committee of the Medical Faculty of the University of Bonn. All scans were acquired during clinical routine and no scan was performed solely for research purposes. The study was conducted in accordance with the Declaration of Helsinki and its amendments.

Patients with multiple dental implants (≥ 5) and associated artefacts receiving a head/neck scan between 12/2021 and 02/2022 were included in this retrospective, single-center study. Implants were located in maxilla and/or mandible. Scans were performed on a clinical PCD-CT (NAEOTOM Alpha, Siemens Healthcare GmbH). Inclusion criteria included age ≥ 18 years, five or more dental implants and the acquisition of a spectral post-processing (SPP) enabled image data set with standard protocol as described below.

**Figure 1:**
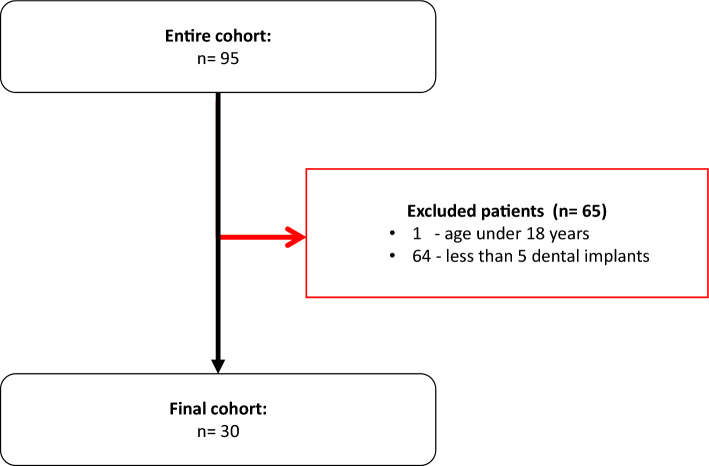
Patient flow chart with exclusion criteria.

Overall, 30 patients were included in the analysis (8 female, 22 male) with an average age of 69 (range: 31–98) years (Fig. 1). Mean DLP was 292.63 mGy*cm and mean CTDI_vol_ was 9.36 mGy.

### Imaging Protocol

A weight-adapted volume of iodine based contrast agent (Accupaque 300 mg/ml, GE Healthcare Buchler GmbH & Co. KG) was applied intravenous with a flow rate of 3 ml/s followed by a bolus of 40 ml of physiologic saline solution. Post-threshold delay was 60 s. Scans were performed in a head-first supine position.

Scan parameters were a tube voltage of 120 kVp with activated automatic tube current modulation, a pitch of 0.8 and a gantry rotation time of 0.5 s. Collimation was 144 × 0.4 mm. Reconstruction parameters were 1.5 mm slice thickness of the reconstruction with an increment of 1 mm. A regular body kernel (Br40; Siemens Healthcare GmbH) as well as Quantum Iterative Reconstruction (QIR Level 3; Siemens Healthcare GmbH, Erlangen, Germany) was used for image reconstruction.

VMI were reconstructed in axial view for 100–190 keV in an interval of 10 keV using dedicated software (syngo.via VB 60, Monoenergetic Plus; Siemens Healthcare GmbH). VMI below 100 keV were not investigated based on adverse results of previous studies^2^.

### Quantitative image analysis

**Figure 2:**
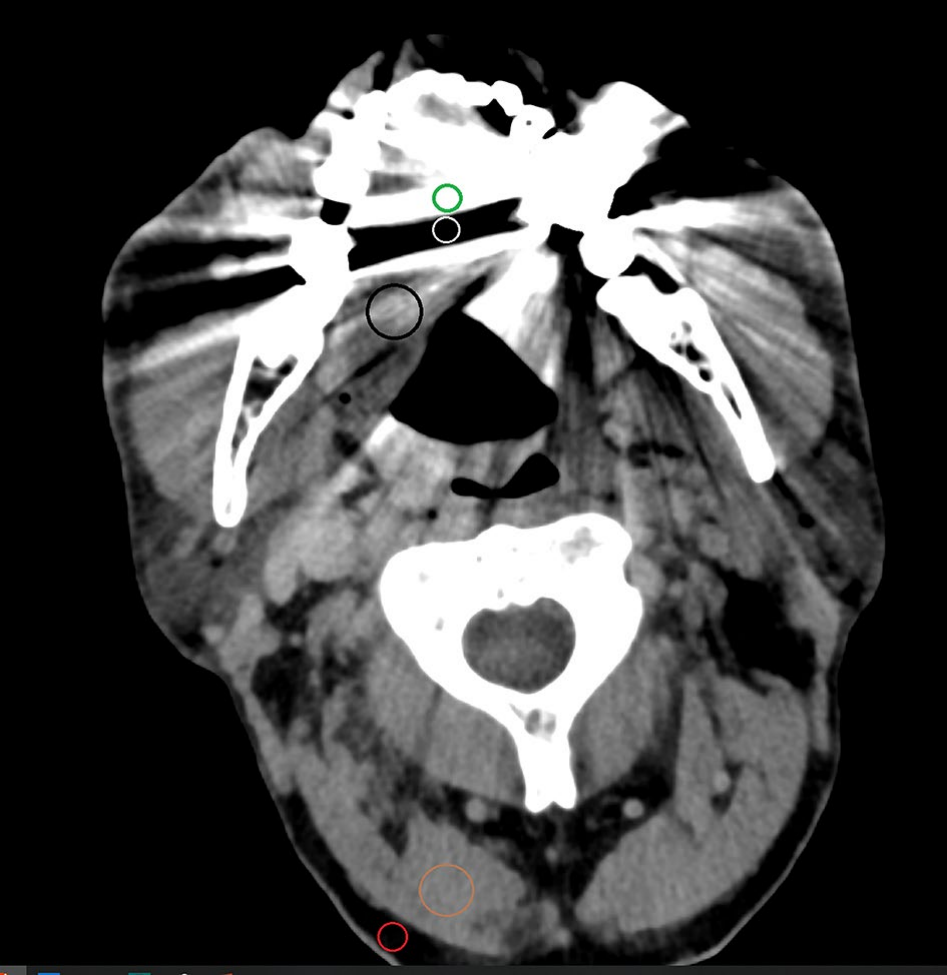
Placement of ROIs (window width/window level 250/50 HU) in the most pronounced hypodense (white) and hyperdense (green) artifacts, in fat tissue (red) and soft tissue areas with (black) and without (brown) presence of artifacts.

Quantitative assessment of polychromatic and virtual monochromatic images was performed using region of interest (ROI) based attenuation analysis in the most pronounced hypodense and hyperdense artifacts using a conventional clinical DICOM viewer (Deep Unity R20 XX; Dedalus HealthCare GmbH). Furthermore, values and standard deviation of X-ray attenuation in fat tissue and soft tissue areas with and without presence of artifacts were evaluated as shown in Fig. 2. Hereby, differences in attenuation and gradient of attenuation of tissue were measured and corrected attenuation and image noise assessed as proposed before^2^. The employed methodology has been established previously and is based on single slices only. The small size of the ROI enables to include only one tissue/anatomical region. Readers were explicitly requested to decide on the slice with the most pronounced hypo- and hyperdense artifacts to avoid bias in image preselection. Therefore ROIs are not necessarily in the same axial level. Similarly, the selected slices do not necessarily show all of the dental implants of a patient, as these can appear very unevenly distributed in mandible and maxilla and readers were free to decide on the chosen slices.

Corrected attenuation was assessed to differ artifact reduction from regular changes in HU for differing VMI energy levels. It was calculated as the difference of muscle tissue impaired by artifacts and without artifact impairment. As image noise is generally higher in images with presence of artifacts, we calculated a corrected image noise as the difference of noise of soft tissue (both muscle and fat) in areas with and without artifacts as proposed before^2,7^. Corrected attenuation of 0 indicates optimal artifact reduction, values above 0 indicate insufficient artifact reduction and values below 0 an overcorrection. All measurements were performed for polychromatic and virtual monochromatic images from 100 to 190 keV in steps of 10 keV.

### Qualitative Image Analysis

Two radiologists with two (YCL) and eleven (DK) years of experience in head and neck CT evaluated the CT images independently regarding artifact extent of hyperdense and hypodense artifacts as well as assessment of soft palate and buccal tissue using a five-point Likert grading scale. The rating of artifacts was defined as follows: (1) excessive artifacts; (2) pronounced artifacts; (3) moderate artifacts; (4) minor artifacts; and (5) artifacts are absent. For assessment of soft palate and buccal tissue the following Likert scale was used: (1) highly restricted diagnostic interpretability; (2) restricted diagnostic interpretability; (3) moderate diagnostic interpretability; (4) minor restrictions on diagnostic interpretability; and (5) unrestricted diagnostic interpretability. For the analysis, the polychromatic image as well as VMI with 100 keV, 130 keV, 160 keV and 190 keV were rated as there are hardly subjective differences in shorter intervals.

### Statistical analysis

All statistical analyses were conducted using IBM SPSS Version 27 (IBM Corp.). Graphs were carried out using the software GraphPad PRISM Version 6.02 (GraphPad Software). Quantitative results are stated as mean and standard deviation. Wilcoxon signed-rank test was used for statistical analysis of quantitative image parameters. Qualitative results are expressed as median with interquartile range (IQR). Interrater reliability was assessed using the intraclass correlation coefficient (ICC). ICC estimates and their 95% confidence intervals (CI) were calculated based on a mean-rating (k = 2), consistency, two-way mixed-effects model^19^. *p*-values below 0.05 were considered significant.

## Results

### Quantitative image analysis

**Figure 3:**
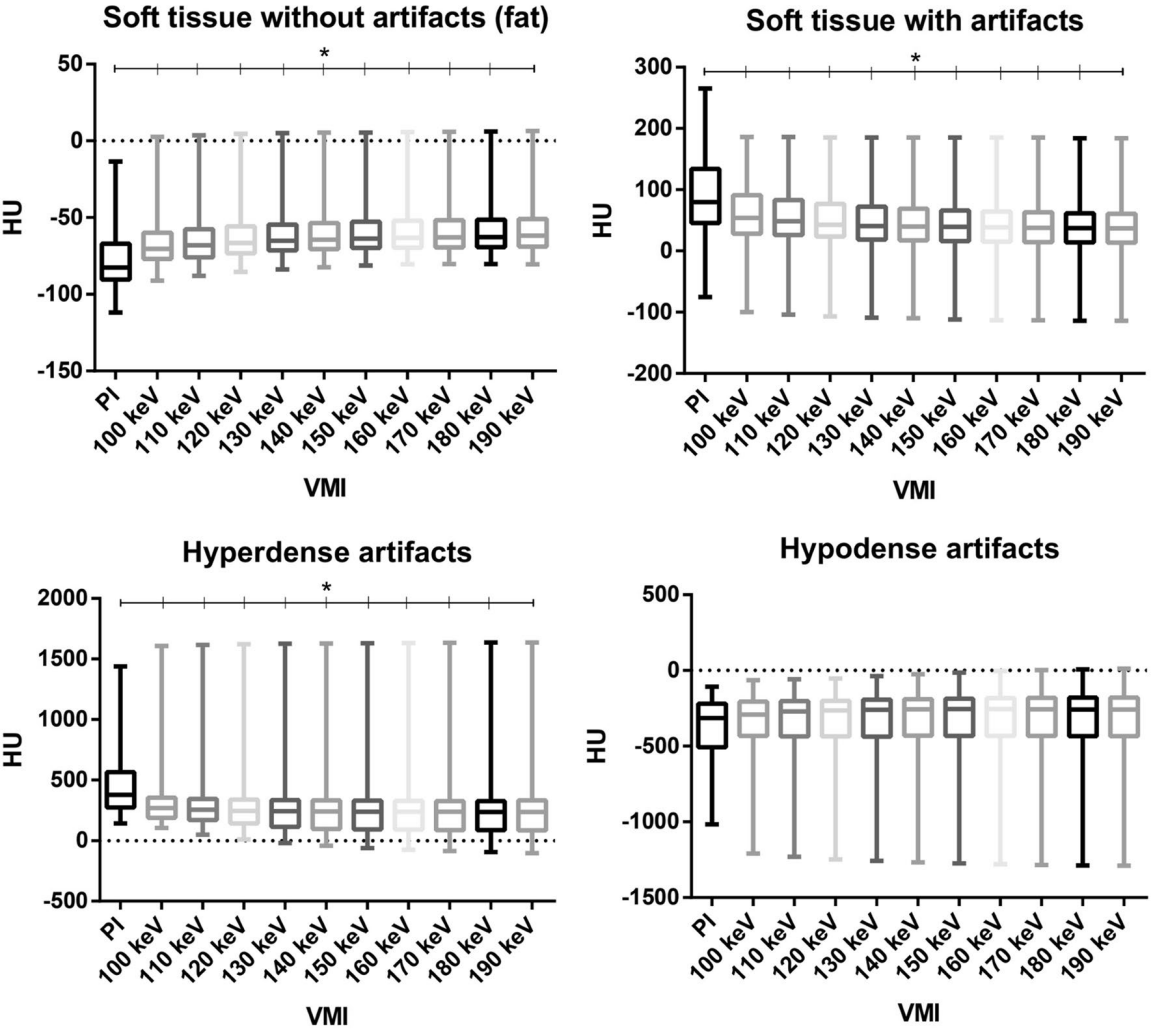
Mean attenuation values within defined regions of interest for soft tissue with presence of artifacts and most pronounced hypodense and hyperdense artifacts. *p*-values below 0.05 are marked with an asterisk.

VMI_130 keV_ showed the best corrected attenuation compared to PI with the closest value to 0 (PI: 30.48 ± 168.64; VMI_130 keV_: − 0.54 ± 110.31; *p* = 0.0026). Corrected attenuation was negative for higher keV VMI ≥ 130 keV, indicating an overcorrection of the artifacts. Mean attenuation of hyperattenuating artifacts decreased in VMI compared to polychromatic images (PI: 474.5 ± 75.9; VMI_130 keV_: 292.5 ± 54; *p* < 0.0001). This effect aggravated with VMI of higher keV (Table 1; Fig. 3). Precise ROI values for CI and each VMI are stated in Table 1.

**Table 1:** Mean attenuation values and standard deviation within defined regions of interest for different reconstructions.

	Hypodense artifacts [HU]	Hyperdense artifacts [HU]	Soft tissue (artifacts) [HU]	Soft tissue (muscle) [HU	Soft tissue (fat) [HU]	Corrected attenuation	Corrected image noise for soft tissue (muscle)	Corrected image noise for soft tissue (fat)
PI	− 387.4 ± 87.10	474.47 ± 75.913	91.97 ± 36.68	61.48 ± 7.34	− 76.67 ± 10.82	30.48 ± 29.34	29.34	138.16
100 keV	− 344.3 ± 74.28 (*p* = 0.1389)	350.10 ± 58.05 (*p* < 0.0001)	59.10 ± 25.43 (*p* < 0.0001)	52.86 ± 5.92 (*p* < 0.0001)	− 65.28 ± 7.96 (*p* < 0.0001)	6.24 ± 19.51 (*p* = 0.0037)	19.51 (*p* < 0.0001)	118.14 (*p* < 0.0001)
110 keV	− 340.1 ± 73.44 (*p* = 0.1746)	334.53 ± 56.55 (*p* < 0.0001)	54.47 ± 24.51 (*p* < 0.0001)	51.53 ± 5.88 (*p* < 0.0001)	− 63.44 ± 7.75 (*p* < 0.0001)	2.95 ± 18.63 (*p* = 0.0026)	18.63 (*p* < 0.0001)	114.97 (*p* < 0.0001)
120 keV	− 337.1 ± 72.74 (*p* = 0.2110)	323.21 ± 55.65 (*p* < 0.0001)	51.82 ± 23.96 (*p* < 0.0001)	50.57 ± 5.88 (*p* < 0.0001)	− 62.07 ± 7.60 (*p* < 0.0001)	1.25 ± 18.08 (*p* = 0.0030)	18.08 (*p* < 0.0001)	112.64 (*p* < 0.0001)
130 keV	− 334.8 ± 72.38 (*p* = 0.2386)	314.68 ± 55.02 (*p* < 0.0001)	49.25 ± 23.58 (*p* < 0.0001)	49.82 ± 5.84 (*p* < 0.0001)	− 61.06 ± 7.51 (*p* < 0.0001)	− 0.55 ± 17.74 (*p* = 0.0026)	17.74 (*p* < 0.0001)	110.87 (*p* < 0.0001)
140 keV	− 333.2 ± 72.09 (*p* = 0.2276)	308.30 ± 54.63 (*p* < 0.0001)	46.60 ± 23.33 (*p* < 0.0001)	49.29 ± 5.84 (*p* < 0.0001)	− 60.31 ± 7.44(*p* < 0.0001)	− 2.69 ± 17.49 (*p* < 0.0037)	17.49 (*p* < 0.0001)	109.59 (*p* < 0.0001)
150 keV	− 331.7 ± 71.80 (*p* = 0.2317)	303.24 ± 54.32 (*p* < 0.0001)	45.09 ± 23.16 (*p* < 0.0001)	48.84 ± 5.87 (*p* < 0.0001)	− 59.71 ± 7.39 (*p* < 0.0001)	− 3.75 ± 17.29 (*p* = 0.0047)	17.29 (*p* < 0.0001)	108.56 (*p* < 0.0001)
160 keV	− 330.8 ± 71.72 (*p* = 0.2778)	299.29 ± 54.17 (*p* < 0.0001)	43.91 ± 23.02 (*p* < 0.0001)	48.52 ± 5.85 (*p* < 0.0001)	− 59.28 ± 7.34 (*p* < 0.0001)	− 4.61 ± 17.17 (*p* = 0.0058)	17.17 (*p* < 0.0001)	107.81 (*p* < 0.0001)
170 keV	− 329.9 ± 71.60 (*p* = 0.2870)	296.28 ± 54.01 (*p* < 0.0001)	43.01 ± 22.92 (*p* < 0.0001)	48.25 ± 5.83 (*p* < 0.0001)	− 58.90 ± 7.30 (*p* < 0.0001)	− 5.25 ± 17.09 (*p* = 0.0059)	17.09 (*p* < 0.0001)	107.15 (*p* < 0.0001)
180 keV	− 329.3 ± 71.46 (*p* = 0.2921)	293.66 ± 53.91 (*p* < 0.0001)	42.20 ± 22.85 (*p* < 0.0001)	48.04 ± 5.84 (*p* < 0.0001)	− 58.61 ± 7.27 (*p* < 0.0001)	− 5.84 ± 17.01 (*p* = 0.0058)	17.01 (*p* < 0.0001)	106.65 (*p* < 0.0001)
190 keV	− 329.5 ± 71.51 (*p* = 0.2965)	292.47 ± 53.98 (*p* < 0.0001)	41.81 ± 22.95 (*p* < 0.0001)	47.86 ± 5.84 (*p* < 0.0001)	− 58.29 ± 7.28 (*p* < 0.0001)	− 6.04 ± 17.11 (*p* = 0.0068)	17.11 (*p* < 0.0001)	106.14 (*p* < 0.0001)

Corrected attenuation closest to 0 shows most favorable artifact reduction. Corrected image noise was calculated for expected lower image noise in high keV and addresses noise without presence of artifacts.

### Qualitative image analysis

**Figure 4:**
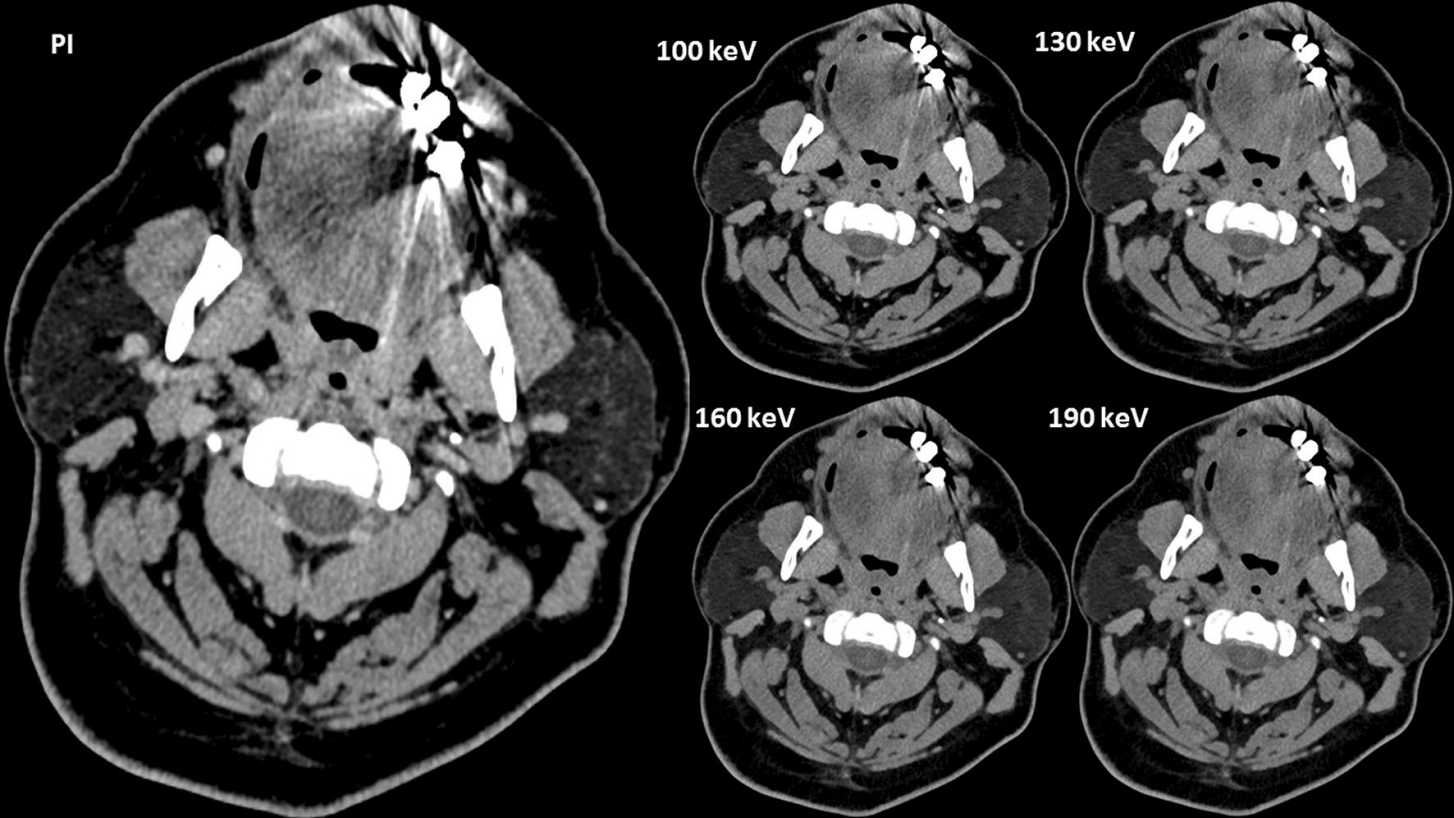
Example of an axial view (window width/window level 250/50 HU) of a conventional polyenergetic reconstruction (PI) compared to virtual monoenergetic images (VMI) with 100 keV, 130 keV, 160 keV and 190 keV.

**Figure 5:**
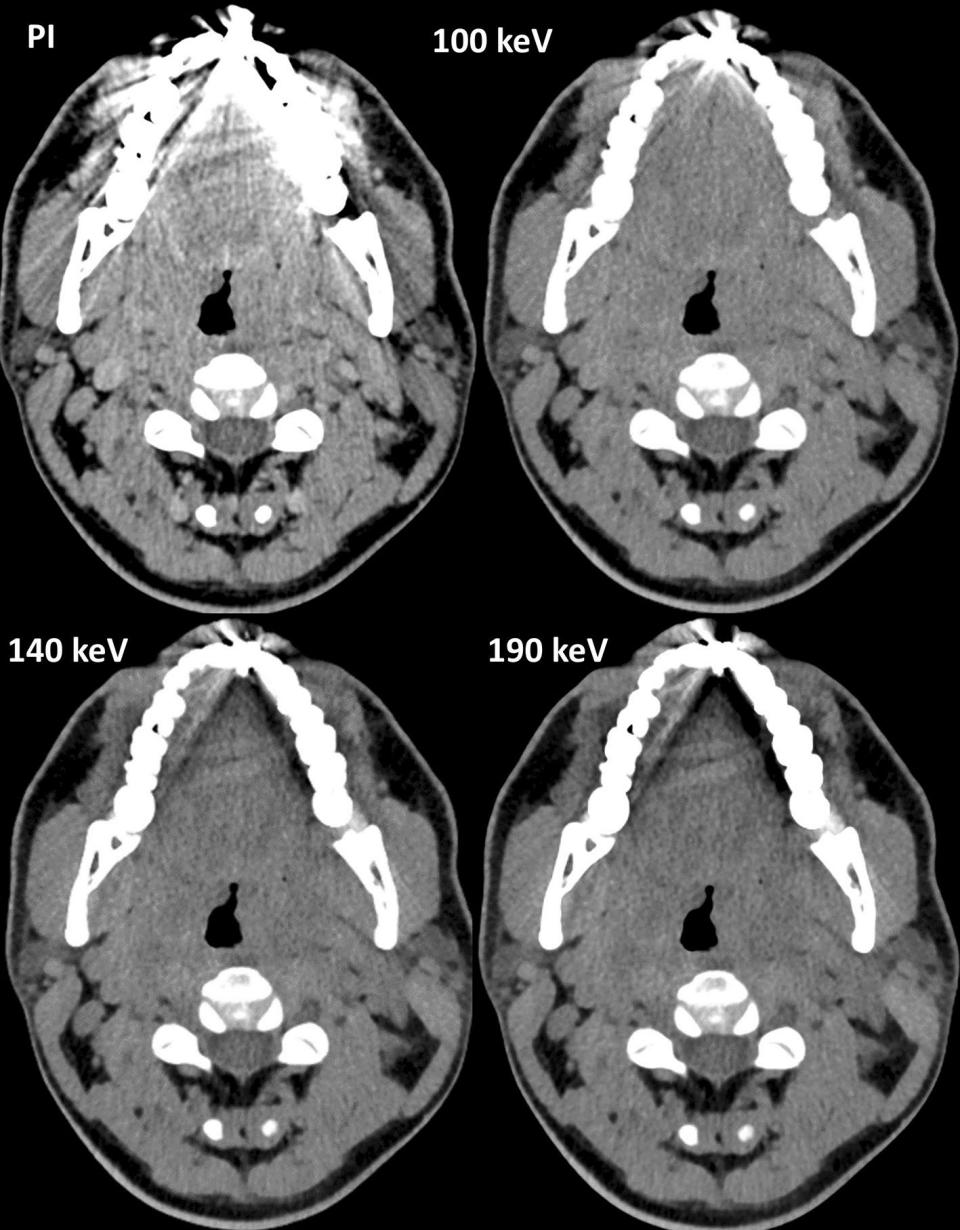
Example of an axial view (window width/window level 250/50 HU) of a conventional polyenergetic reconstruction (PI) and virtual monoenergetic images (VMI) with 100 keV, 120 keV and 140 keV. With higher keV increasing hypodense streaks occur.

**Figure 6:**
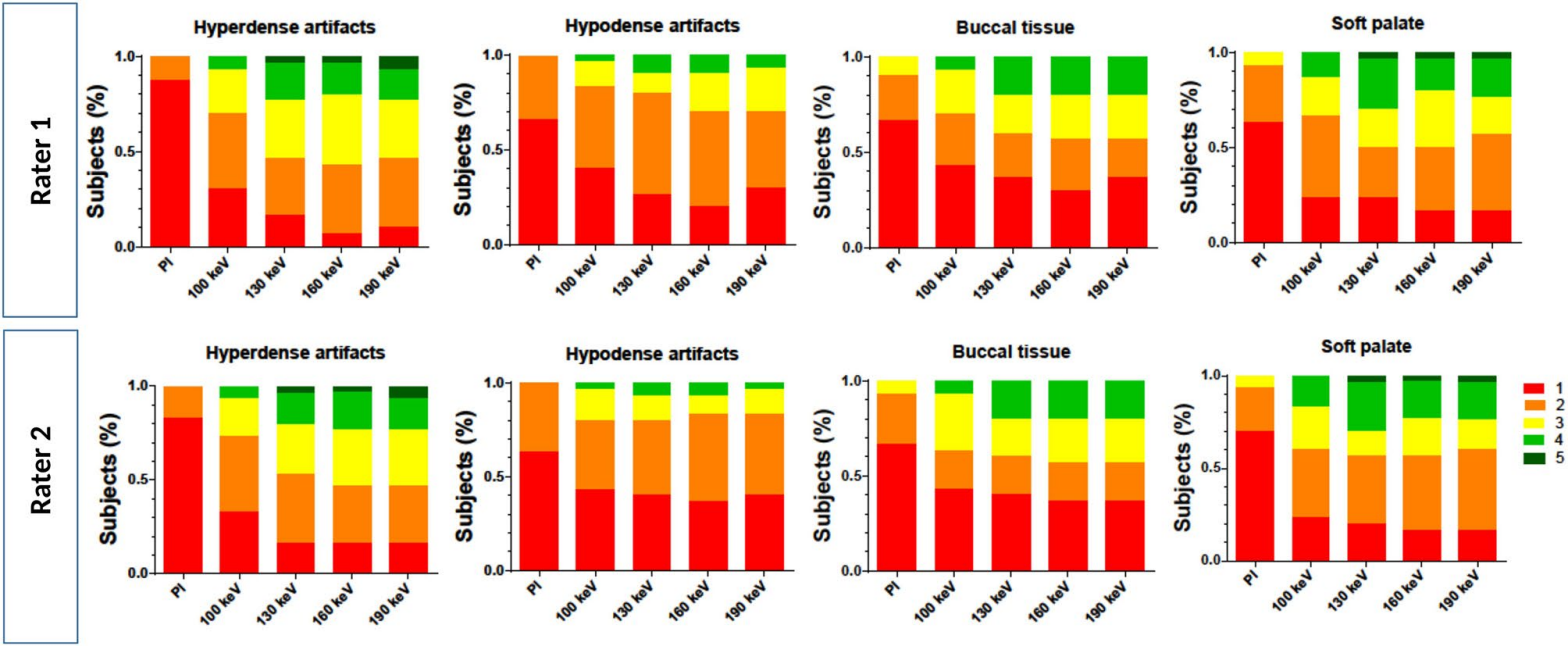
Bar plots show distribution of hypodense artifact extent, hyperdense artifact extent, soft palate diagnostic quality and buccal diagnostic quality rating of polyenergetic reconstruction (PI) and virtual monoenergetic images (VMI) with 100 keV, 130 keV, 160 keV and 190 keV.

**Figure 7:**
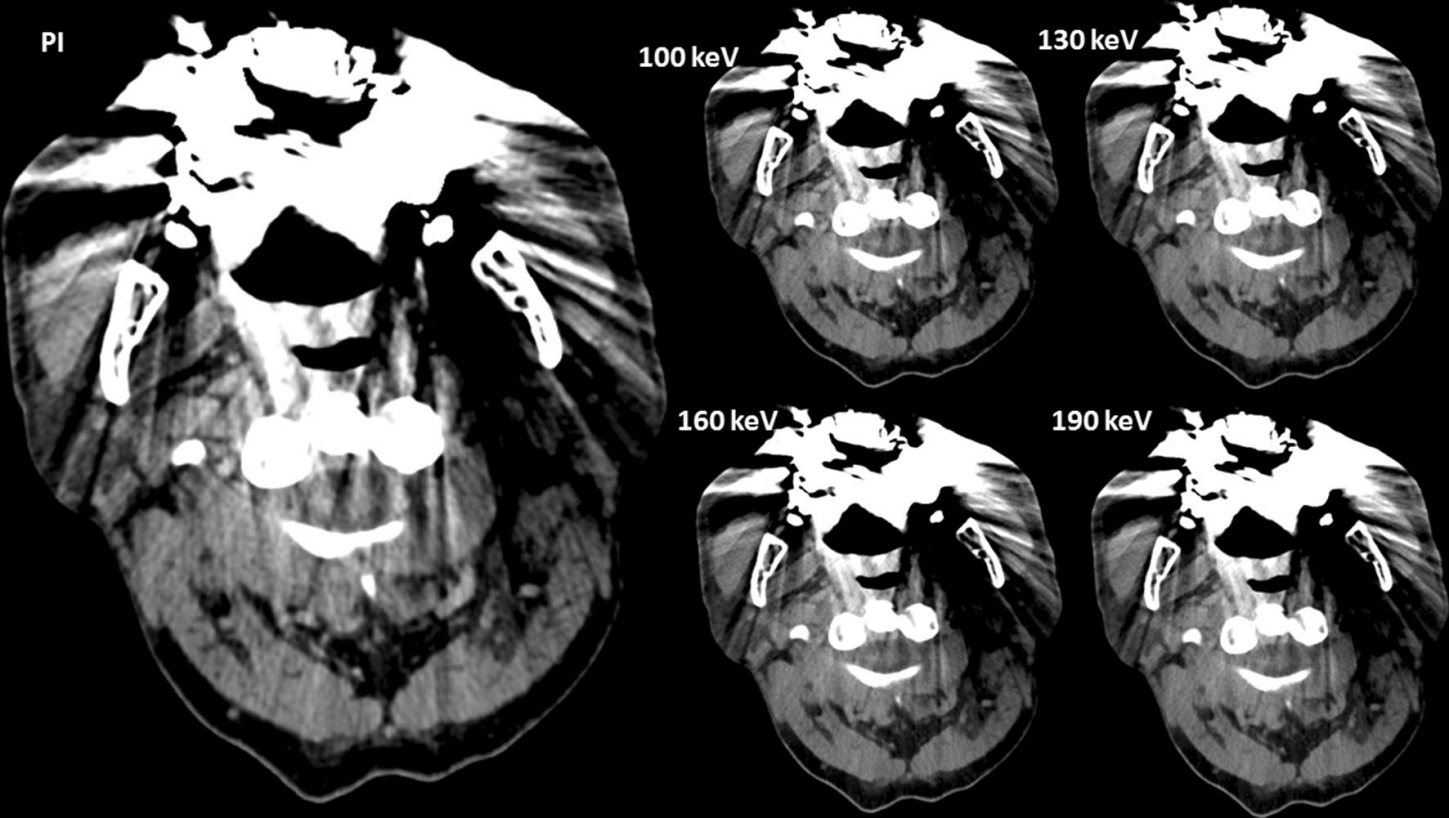
Example of an axial view (window width/window level 250/50 HU) of a conventional polyenergetic reconstruction (PI) compared to virtual monoenergetic images (VMI) with 100 keV, 130 keV, 160 keV and 190 keV. Regarding these most excessive artifacts only little visible improvements occur, regardless of keV.

Qualitative assessment of soft palate (SP) and buccal tissue (BT) improved compared to conventional image (Median PI: SP 1 (1–2); BT 1 (1–2); Median VMI_130 keV_: SP 2 (2–4), BT 2 (1–3); *p* < 0.0001; Supplementary Information 1). Hyperdense artifacts were subjectively reduced, scoring a 2.5 (2–3; *p* < 0.0001) at 130 keV. For the PI the score was 1 (1–1) and VMI_190 keV_ scored 3 (2–3; *p* < 0.0001). For hypodense artifacts, PI scored 1 (1–2) and VMI_130 keV_ scored 2(1–2; *p* < 0.0001). No remarkable difference was observed between VMI_160 keV_ and VMI_190 keV_ (Fig. 4). In some cases hypodense artifacts increased with higher keV VMI (Fig. 5.). Rating results for each criteria by each rater are shown in Fig. 6. Regarding the most pronounced artifacts, only small improvements could be achieved, regardless of the keV, as shown in Fig. 7.

Interrater agreement was excellent with an overall ICC value of 0.973 (95% confidence interval: 0.968; 0.977). Respective ICC was 0.933 (95% CI 0.907–0.951) for the extent of hypodense artifacts, 0.983 (95% CI 0.976–0.987) for the extent of hyperdense artifacts, 0.978 (95% CI 0.969–0.984) for assessment of the buccal tissue and 0.980 (95% CI 0.972–0.985) for assessment of the soft palate.

## Discussion

This study evaluates the impact of virtual monoenergetic image reconstruction on artifacts from dental implants in photon counting detector CT. The current results indicate that VMI are a useful tool to reduce dental implant artifacts and, thus enable a better assessment of the oral cavity. VMI associated image quality improvement may enable detection of pathologies that would otherwise be concealed by artifacts in conventional CT images.

There are different causes of implant-associated artifacts. Beam hardening, based on the preferential signal-loss of low-energy photons, causes streaking hypodense artifacts^20^. Photon starvation, which leads to hypodense artifacts, is observed when too few photons can pass a dense structure^21^. Scatter artifacts, mostly due to incoherent scattering, contribute to a higher X-ray attenuation^22,23^. High energetic VMIs mainly address the artifacts caused by beam hardening. As low-energy photons are absorbed more rapidly by an object they pass, the mean detected photon energy increases, causing artifacts during image reconstruction^24^. With the use of high keV VMI, these artifacts can be reduced. High keV images, however, typically show a decrease in contrast. CNR in PCD-CT is higher than in EID-CT^15,25–27^, thus PCD-CT technology allows for higher keV reconstructions in diagnostic assessment.

The effect of VMI associated artifact reduction reached a plateau at VMI_160 keV_, no further improvement was seen at higher energetic reconstructions. A possible explanation for this phenomenon is that the beneficial effects of VMI are already maximized at VMI_160 keV_.

In the current study, artifacts were not completely eliminated in VMI but merely decreased. These remaining artifacts might be caused by other effects than beam hardening, for instance photon starvation. Another possible explanation is the selection of patients with at least five dental implants and therefore comparatively extensive artifacts. Supporting this thesis, previous studies with extensive artifacts observed only minor differences especially between VMI with high keV^2^.

The extent of both hypodense and hyperdense artifacts was reduced in reconstructed VMI which resulted in an improvement of qualitative and quantitative scores. For hyperdense artifacts, the reduction of artifact extent as well as the increase of image quality was highly significant. Although the same tendencies were seen for hypodense artifacts, results were not significant, analogue to studies previously conducted on EID-CT^2,5,7^.

For quantitative assessment, the method of placement of ROIs for measurement of HU to quantify the extent of artifacts was used. There are various other approaches to determine the extent of artifacts, including voxel measurements and calculation formula^28,29^. There is currently no consensus regarding a gold standard for the assessment of artifact magnitude in CT. In the current study, a practical and comprehensible method was employed. For qualitative assessment, two radiologists independently scored images using a Likert-scale grading system ranging from one to five, with one being the worst quality and five the best achievable quality. An analogous approach was also employed in previous similar studies^2,7^.

Important differences in artifact reduction seem to be caused by material composition and material orientation, as it in general determines the extent of artifacts^21^. The optimal keV setting will most likely depend on material that caused the artifact as well as the extent of artifacts. Although a personalized algorithm taking the specific implant material of each patient into account would be desirable, clinical implementation is not feasible. Thus, a universal solution is necessary for clinical practice. In some rare cases, we experienced new hypodense streaks that aggravate with increasing keV and thus worsen diagnostic quality of the region. This phenomenon could not be observed for most dental implants and fillings. In some cases, there was hardly a difference between subjective impressions of the artifacts between the different VMI, while quantitively difference could be shown to the PI. Overall, for most cases, the best diagnostic quality was observed at 130 keV. Given that, the recommendation of the authors would be to additionally reconstruct VMIs at 130 keV for PCD-CT examinations with extensive dental implant associated artifacts. The results of our studies are in line with previous studies for reduction of dental implant associated artifacts performed on a dual layer dual energy CT, favoring a keV setting between 130 and 160 keV^2,7^. Studies performed on dual source dual energy CT recommend a slightly lower keV between 100 and 130 keV, especially due to the decrease of contrast at higher keV^30,31^. In general, most studies favor a VMI energy level between 100 and 140 keV^4,32–36^. A first phantom study on PCD-CT stated no benefit from higher-energetic reconstructions, contrary to our results^37^. A recent study which assessed artifacts of cochlear implants in both PCD-CT and EID-CT, found a comparable artifact reduction for VMI of EID-CT and PCD-CT^38^.

There are several limitations to this study. Study setup was retrospective, single-centered and only included a small number of patients. Further studies with more patients included are necessary to test clinical benefit of VMI, especially once additional artifact reduction techniques are clinically available and can be compared.

Caused by the retrospective character of the study, no exact dental history was available. Therefore, the various composition of the included implants is unknown. However, this resembles a real life situation, where there is usually no knowledge available regarding the material of dental implants. Nevertheless, as different materials cause different artifacts, it would be of interest to what extent the artifacts of each material can be reduced using VMI^39^. We noticed that the extent of artifacts differed vastly depending on the type of implant. Thus, studies investigating optimal artifact reconstruction depending on implant type are needed.

There is the need for further studies on PCD-CT to address the performance of metal artifact reduction algorithms (MAR) also in combination with VMI. At the time of the end of the investigation, there was no clinical MAR for the PCD-CT available. Even though VMI showed improvements in diagnostic image quality, artifacts were merely reduced, not eliminated. There is still potential to further optimize artifact reduction for extended artifacts.

## Conclusion

In summary, the current results show that VMI lower dental—implant associated artifacts of the craniomandibular region in PCD-CT images. VMI reconstructed at 130 keV showed the best results as a tradeoff between artifact reduction and image quality. Higher kev reconstructions lead to lower contrast and the appearance of extensive, new hypodense artifacts.

###  Supplementary Information

Supplementary Table 1.

## Data Availability

The anonymized datasets generated during and analyzed during the current study are available from the corresponding author on reasonable request. Due to local privacy laws, CT images can not be provided as theoretically there is a risk of identification of personal information in pseudo-anonymized CT datasets.

## References

[1] Brandfass JT, Ulano AC, Nickerson JP, Bazylewicz MP (2019). Dental caries on CT in the ER population: Prevalence and reporting practices. Emerg. Radiol..

[2] Laukamp KR (2019). Metal artifacts in patients with large dental implants and bridges: Combination of metal artifact reduction algorithms and virtual monoenergetic images provides an approach to handle even strongest artifacts. Eur. Radiol..

[3] Barrett JF, Keat N (2004). Artifacts in CT: Recognition and avoidance. Radiographics.

[4] de Crop A (2015). Analysis of metal artifact reduction tools for dental hardware in CT scans of the oral cavity: kVp, iterative reconstruction, dual-energy CT, metal artifact reduction software—Does it make a difference?. Neuroradiology.

[5] Schmidt AMA (2022). Combination of iterative metal artifact reduction and virtual monoenergetic reconstruction using split-filter dual-energy CT in patients with dental artifact on head and neck CT. AJR Am. J. Roentgenol..

[6] Wei Y (2020). Clinical application of multi-material artifact reduction (MMAR) technique in Revolution CT to reduce metallic dental artifacts. Insights Imaging.

[7] Große Hokamp N (2018). Artifact reduction from dental implants using virtual monoenergetic reconstructions from novel spectral detector CT. Eur. J. Radiol..

[8] D'Angelo T (2019). Dual energy computed tomography virtual monoenergetic imaging: Technique and clinical applications. Br. J. Radiol..

[9] Willemink MJ, Grist TM (2022). Counting photons: The next era for CT imaging?. Radiology.

[10] Kämmerling N, Sandstedt M, Farnebo S, Persson A, Tesselaar E (2022). Assessment of image quality in photon-counting detector computed tomography of the wrist—An ex vivo study. Eur. J. Radiol..

[11] Rajendran K (2023). Improved visualization of the wrist at lower radiation dose with photon-counting-detector CT. Skelet. Radiol..

[12] Grunz J-P (2022). Spectral shaping via tin prefiltration in ultra-high-resolution photon-counting and energy-integrating detector CT of the temporal bone. Investig. Radiol..

[13] Wrazidlo R (2022). Radiation dose reduction in contrast-enhanced abdominal CT: Comparison of photon-counting detector CT with 2nd generation dual-source dual-energy CT in an oncologic cohort. Acad. Radiol..

[14] Woeltjen MM (2022). Low-dose high-resolution photon-counting CT of the lung: Radiation dose and image quality in the clinical routine. Diagnostics.

[15] Hagen, F. *et al.* Image Quality and Radiation Dose of Contrast-Enhanced Chest-CT Acquired on a Clinical Photon-Counting Detector CT vs. Second-Generation Dual-Source CT in an Oncologic Cohort: Preliminary Results. *Tomography (Ann Arbor, Mich.)* **8,** 1466–1476 (2022). 10.3390/tomography8030119 PMC9227736 35736867

[16] Grunz J-P (2022). Ultra-Low-Dose Photon-Counting CT Imaging of the Paranasal Sinus With Tin Prefiltration: How Low Can We Go?. Investigative radiology.

[17] Hsieh SS, Leng S, Rajendran K, Tao S, McCollough CH (2021). Photon Counting CT: Clinical Applications and Future Developments. IEEE transactions on radiation and plasma medical sciences.

[18] Zhou W (2019). Reduction of metal artifacts and improvement in dose efficiency using photon-counting detector computed tomography and tin filtration. Investig. Radiol..

[19] Koo TK, Li MY (2016). A guideline of selecting and reporting intraclass correlation coefficients for reliability research. J. Chiropr. Med..

[20] Lee M-J (2007). Overcoming artifacts from metallic orthopedic implants at high-field-strength MR imaging and multi-detector CT. Radiographics.

[21] Kalisz K (2016). Artifacts at cardiac CT: Physics and solutions. Radiographics.

[22] Bushberg JT (1998). The AAPM/RSNA physics tutorial for residents. X-ray interactions. Radiographics.

[23] Mori I, Machida Y, Osanai M, Iinuma K (2013). Photon starvation artifacts of X-ray CT: Their true cause and a solution. Radiol. Phys. Technol..

[24] Pessis E (2013). Virtual monochromatic spectral imaging with fast kilovoltage switching: reduction of metal artifacts at CT. Radiographics.

[25] Higashigaito K (2022). Contrast-enhanced abdominal CT with clinical photon-counting detector CT: Assessment of image quality and comparison with energy-integrating detector CT. Acad. Radiol..

[26] Hagen F (2022). Image quality and dose exposure of contrast-enhanced abdominal CT on a 1st generation clinical dual-source photon-counting detector CT in obese patients vs. a 2nd generation dual-source dual energy integrating detector CT. Eur. J. Radiol..

[27] Graafen D (2022). Photon-counting detector CT improves quality of arterial phase abdominal scans: A head-to-head comparison with energy-integrating CT. Eur. J. Radiol..

[28] Pomerantz SR (2013). Virtual monochromatic reconstruction of dual-energy unenhanced head CT at 65–75 keV maximizes image quality compared with conventional polychromatic CT. Radiology.

[29] Große Hokamp N (2018). Reduction of artifacts caused by deep brain stimulating electrodes in cranial computed tomography imaging by means of virtual monoenergetic images, metal artifact reduction algorithms, and their combination. Investig. Radiol..

[30] Stolzmann P (2013). Monoenergetic computed tomography reconstructions reduce beam hardening artifacts from dental restorations. Forensic Sci. Med. Pathol..

[31] Roele ED, Timmer VCML, Vaassen LAA, van Kroonenburgh AMJL, Postma AA (2017). Dual-energy CT in head and neck imaging. Curr. Radiol. Rep..

[32] Bamberg F (2011). Metal artifact reduction by dual energy computed tomography using monoenergetic extrapolation. Eur. Radiol..

[33] Guggenberger R (2012). Metallic artefact reduction with monoenergetic dual-energy CT: Systematic ex vivo evaluation of posterior spinal fusion implants from various vendors and different spine levels. Eur. Radiol..

[34] Takrouri HS (2015). Metal artifact reduction: Added value of rapid-kilovoltage-switching dual-energy CT in relation to single-energy CT in a piglet animal model. Am. J. Roentgenol..

[35] Wang Y (2013). Metal artifacts reduction using monochromatic images from spectral CT: Evaluation of pedicle screws in patients with scoliosis. Eur. J. Radiol..

[36] Zhou C (2011). Monoenergetic imaging of dual-energy CT reduces artifacts from implanted metal orthopedic devices in patients with factures. Acad. Radiol..

[37] Julian A (2022). Iterative metal artifact reduction on a clinical photon counting system—technical possibilities and reconstruction selection for optimal results dependent on the metal scenario. Phys. Med. Biol.

[38] Waldeck S (2022). Photon-counting detector CT virtual monoengergetic images for cochlear implant visualization-A head to head comparison to energy-integrating detector CT. Tomography.

[39] Klinke T (2012). Artifacts in magnetic resonance imaging and computed tomography caused by dental materials. PloS One.

