# Supplementary material for: Use of virtual monoenergetic images for reduction of extensive dental implant associated artifacts in photon-counting detector CT
Source: Sci Rep. 2024 Jan 4;14:497. doi: 10.1038/s41598-023-50926-3 (PMC10766624; doi:10.1038/s41598-023-50926-3)
Supplement: Supplementary file 1 — Supplementary Table 1. [file 41598_2023_50926_MOESM1_ESM.pdf]

	PI (range)	VMI 100 keV (range)	VMI 130 keV (range)	VMI 160 keV (range)	VMI 190 keV (range)	ICC
Extent of hypodense artifacts	1 (1-2)	2 (1-4) ($p < 0.0001$)	2 (1-4) ($p < 0.0001$)	2 (1-4) ($p < 0.0001$)	2 (1-4) ($p < 0.0001$)	0.933 (0.951; 0.951)
Extent of hyperdense artifacts	1 (1-2)	2 (1-4) ($p < 0.0001$)	2.5 (1-5) ($p < 0.0001$)	3 (1-5) ($p < 0.0001$)	3 (1-5) ($p < 0.0001$)	0.983 (0.976; 0.987)
Diagnostic quality of soft palate	1 (1-3)	2 (1-4) ($p < 0.0001$)	2 (1-5) ($p < 0.0001$)	2 (1-5) ($p < 0.0001$)	2 (1-5) ($p < 0.0001$)	0.978 (0.969; 0.984)
Diagnostic quality of buccal tissue	1 (1-3)	2 (1-4) ($p < 0.0001$)	2 (1-4) ($p < 0.0001$)	2 (1-4) ($p < 0.0001$)	2 (1-4) ($p < 0.0001$)	0.980 (0.972; 0.985)

Supplementary Information 1: Median and Range of qualitative image ratings. Intraclass correlation coefficient (ICC) estimates and their 95% confident intervals were calculated. ICC calculation is based on a mean-rating (k=2), consistency, two-way mixed-effects model.
